# Chemical-Physical Properties and Bioactivity of New Premixed Calcium Silicate-Bioceramic Root Canal Sealers

**DOI:** 10.3390/ijms232213914

**Published:** 2022-11-11

**Authors:** Fausto Zamparini, Carlo Prati, Paola Taddei, Andrea Spinelli, Michele Di Foggia, Maria Giovanna Gandolfi

**Affiliations:** 1 Endodontic Clinical Section, School of Dentistry, Department of Biomedical and Neuromotor Sciences, University of Bologna, 40125 Bologna, Italy; 2 Laboratory of Green Biomaterials and Oral Pathology, School of Dentistry, Department of Biomedical and Neuromotor Sciences, University of Bologna, 40125 Bologna, Italy; 3 Biochemistry Unit, Department of Biomedical and Neuromotor Sciences, University of Bologna, 40126 Bologna, Italy

**Keywords:** endodontic sealers, root canal sealers, calcium silicates, calcium silicates cements, bioceramics, bioactivity, calcium phosphate nucleation, apatite nucleation

## Abstract

The aim of the study was to analyze the chemical–physical properties and bioactivity (apatite-forming ability) of three recently introduced premixed bioceramic root canal sealers containing varied amounts of different calcium silicates (CaSi): a dicalcium and tricalcium silicate (1–10% and 20–30%)-containing sealer with zirconium dioxide and tricalcium aluminate (CERASEAL); a tricalcium silicate (5–15%)-containing sealer with zirconium dioxide, dimethyl sulfoxide and lithium carbonate (AH PLUS BIOCERAMIC) and a dicalcium and tricalcium silicate (10% and 25%)-containing sealer with calcium aluminate, tricalcium aluminate and tantalite (NEOSEALER FLO). An epoxy resin-based sealer (AH PLUS) was used as control. The initial and final setting times, radiopacity, flowability, film thickness, open pore volume, water absorption, solubility, calcium release and alkalizing activity were tested. The nucleation of calcium phosphates and/or apatite after 28 days aging in Hanks balanced salt solution (HBSS) was evaluated by ESEM-EDX, vibrational IR and micro-Raman spectroscopy. The analyses showed for NeoSealer Flo and AH Plus the longest final setting times (1344 ± 60 and 1300 ± 60 min, respectively), while shorter times for AH Plus Bioceramic and Ceraseal (660 ± 60 and 720 ± 60 min, respectively). Radiopacity, flowability and film thickness complied with ISO 6876/12 for all tested materials. A significantly higher open pore volume was observed for NeoSealer Flo, AH Plus Bioceramic and Ceraseal when compared to AH Plus (*p* < 0.05), significantly higher values were observed for NeoSealer Flo and AH Plus Bioceramic (*p* < 0.05). Ceraseal and AH Plus revealed the lowest solubility. All CaSi-containing sealers released calcium and alkalized the soaking water. After 28 days immersion in HBSS, ESEM-EDX analyses revealed the formation of a mineral layer that covered the surface of all bioceramic sealers, with a lower detection of radiopacifiers (Zirconium for Ceraseal and AH Plus Bioceramic, Tantalum for NeoSealer Flo) and an increase in calcium, phosphorous and carbon. The calcium phosphate (CaP) layer was more evident on NeoSealer Flo and AH Plus Bioceramic. IR and micro-Raman revealed the formation of calcium carbonate on the surface of all set materials. A thin layer of a CaP phase was detected only on AH Plus Bioceramic and NeoSealer Flo. Ceraseal did not show CaP deposit despite its highest calcium release among all the tested CaSi-containing sealers. In conclusion, CaSi-containing sealers met the required chemical and physical standards and released biologically relevant ions. Slight/limited apatite nucleation was observed in relation to the high carbonation processes.

## 1. Introduction

Since their first application as materials for root-end surgery, calcium silicate-based materials demonstrated excellent sealing ability and were able to set in the presence of moisture (such as blood or saliva) [1,2,3,4,5].

Calcium silicate-based materials (i.e., mainly containing CaSi particles) possess high biocompatibility and favorable biological properties, as demonstrated in a number of in vitro (cells studies) [6,7] and ex vivo (animal models) studies [8,9,10]. These positive interactions with biological tissues have been mostly attributed to their release of biologically interactive ions (such as calcium) [11,12,13,14] and the nucleation of an apatite layer on their surface [15,16,17], which starts immediately after the material hydration [18,19]. For these properties, calcium silicate-based materials have found a pivotal role in approaching complex endodontic cases, namely perforation repair and apical plugs for teeth with open apexes [1,2,3,4,5]. The first generation of calcium silicate-based materials demonstrated some limitations, mostly attributable to their long setting time, low radiopacity, handling difficulties and grayish discoloration, which restricted the use of these materials as root canal sealers [20,21]. Modification of these endodontic materials to overcame most of these limitations have been done and root canal sealers based on calcium silicates have been proposed 10–15 years ago as powder–liquid or paste-to-paste formulations [3,5,22].

In recent times, premixed flowable sealers have been introduced for root canal treatment. Differently from the other formulations, these materials are ready to be used and do not require mixing as their setting reaction is achieved in the presence of moisture. Lately, CaSi-based materials have been generically named “bioceramics”. It must be pointed out that the definition of ceramic is generic and non-specific, referring to an inorganic material constituted by the combination of metallic and non-metallic elements. The term “bioceramics”, coined to highlight the positive biological behavior, refers to ceramic materials used for repairing or replacing damaged bone tissues. Bioceramics can directly interact with the surrounding tissue, either supporting tissue growth or inducing new tissue regeneration [23]. Therefore, the term “bioceramic” is vague and does not specifically refers to CaSi-containing materials. A positive interaction with surrounding periapical tissues without inducing inflammation or foreign body reactions has been reported for calcium silicate-based materials [8,9,10], while no studies exist regarding some recently introduced materials.

These new bioceramics have been developed by adding different percentages of CaSi and different radiopacifiers in their composition.

In this context, we want to highlight a distinction between calcium silicate-based (i.e., mainly containing CaSi particles) and calcium silicate-containing (i.e., containing minor amounts of CaSi) sealers.

Ceraseal is a premixed bioceramic sealer including tricalcium silicate (20–30%) and dicalcium silicate (1–10%) as bioactive components, and tricalcium aluminate (1–10%) and zirconium dioxide (45–50%) as radiopacifiers. Some traces of thickening agents are reported by the manufacturer.

NeoSealer Flo is a premixed bioceramic sealer constituted by tricalcium silicate (<25%) and dicalcium silicate (<10%) as bioactive components, and calcium aluminate (<25%), calcium aluminum oxide (grossite) (<6%), tricalcium aluminate (<5%) and tantalite (50%) as radiopacifier. Traces of calcium sulfate (<1%) are also reported by the manufacturer.

AH Plus Bioceramic is a premixed bioceramic sealer mostly composed of zirconium dioxide (50–70%) as a radiopacifier and tricalcium silicate (10–15%) as a bioactive component. Dimethyl sulfoxide and traces of lithium carbonate and thickening agents are also reported by the manufacturer.

To date, no literature is available regarding the chemical–physical properties and bioactivity of these recent bioceramic root canal sealers. Therefore, the aim of the study was to evaluate different clinically relevant chemical–physical properties such as ion release, setting times, radiopacity, open pore volume, water absorption, solubility, flow and film thickness. The ability to nucleate apatite has also been assessed. The sealers were compared to a traditional epoxy resin-based sealer (AH Plus) used as control.

## 2. Results

### 2.1. Initial, Final Setting Times and Radiopacity

Setting times are reported in Table 1. Ceraseal showed the shortest initial setting times (60 ± 5 min), while NeoSealer Flo showed the longest setting time (480 ± 125 min). Ceraseal and AH Plus Bioceramic had similar final setting times (660 ± 60 and 720 ± 60 min), while NeoSealer Flo and AH Plus revealed significantly longer final setting time values (1300 ± 60 and 1344 ± 60 min) (*p* < 0.05). All sealers complied with ISO 6876/12: they showed different radiopacity values, but always higher than 3.0 mmAl. In particular, NeoSealer Flo showed the lowest value (5.0 ± 0.5 mmAl), and AH Plus the highest (11.5 ± 0.5 mmAl). AH Plus Bioceramic and Ceraseal showed intermediate values (8.0 ± 0.5 and 8.6 ± 0.5 mmAl, respectively).

### 2.2. Sealer Flowability and Film Thickness

Flowability and film thickness are reported in Table 2. Ceraseal showed the highest flowability and the lowest film thickness values, while NeoSealer Flo revealed the lowest flowability and highest film thickness values. AH Plus Bioceramic showed intermediate values. All sealers complied with ISO 6876/2012.

### 2.3. Open Pore Volume, Water Absorption and Solubility

Table 3 reports the volume of open pores, water absorption and solubility of the tested materials. NeoSealer Flo and AH Plus Bioceramic showed higher volumes of open pores (0.048 ± 0.011 and 0.042 ± 0.011, respectively) when compared to Ceraseal. AH Plus showed similar solubility when compared to Ceraseal (*p* > 0.05), but significantly lower porosity and water absorption (*p* < 0.05).

### 2.4. Alkalizing Activity and Calcium Release

All the calcium silicate-based materials provided alkalization of the soaking medium and released calcium (Table 4 and Table 5).

Until 14 days, Ceraseal provided the highest pH values while NeoSealer Flo and AH Plus Bioceramic sealer provided lower alkalizing activity; at 28 days, the three sealers show comparable pH values. AH Plus did not alkalize the environment, a slight acidification of soaking water was observed from 1-day immersion.

The sealers that contained a higher amount of CaSi had higher cumulative Ca release. Ceraseal provided the highest values. NeoSealer Flo showed lower calcium release, with significant reduction for 14 days. AH Plus Bioceramic sealer proved high calcium release values after 3 and 7 days; the values reduced at the subsequent endpoints. AH Plus did not release calcium ions.

### 2.5. Surface Characterization and CaP Nucleation

#### 2.5.1. Ceraseal

**Figure 1:**
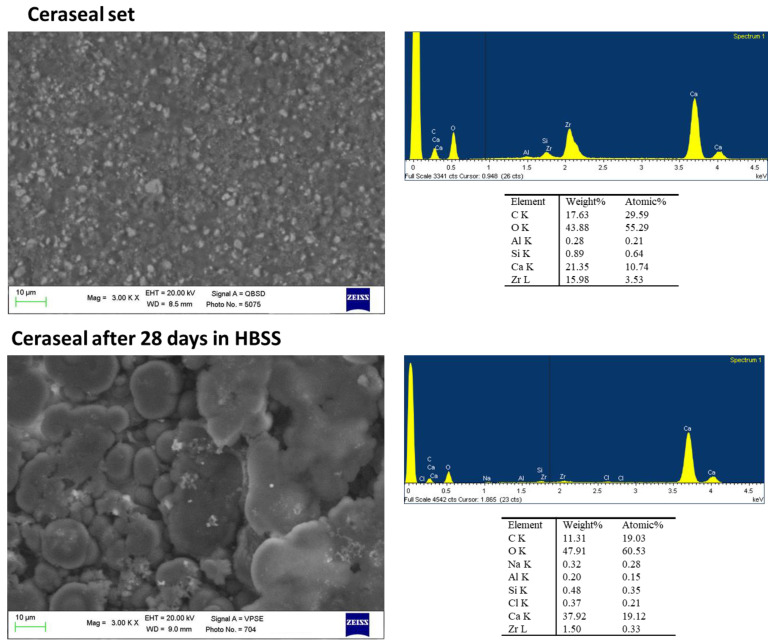
ESEM images at 3000× of Ceraseal before and after immersion in HBSS. Set Ceraseal sample was characterized by a regular surface with small granules widely spread. EDX revealed the constitutional elements of the materials, namely Zr, Ca, Si and Al. After 28 days immersion, an irregular surface was observed. Numerous globular structures covering the sealer surface were detected. EDX revealed an increase in Ca and a decrease in Zr and Si. No P was detected.

The set surface of Ceraseal was observed through ESEM at 3000× magnification. The surface was regular with small granules (range 2–5 µm) widely distributed. EDX revealed the constitutional elements of the material, as declared by the manufacturer, namely zirconium (the radiopacifier used in this formulation), calcium and silicon (from calcium silicates). Al was detected as well (Figure 1). After 28 days immersion, ESEM images revealed an irregular surface with well-evident globular structures covering the sealer surface (ranging from 10 to 20 µm). EDX revealed an increase in Ca and a decrease in Zr and Si. Sodium (Na) and chlorine (Cl) were attributable to the HBSS solution. No P was detected.

**Figure 2:**
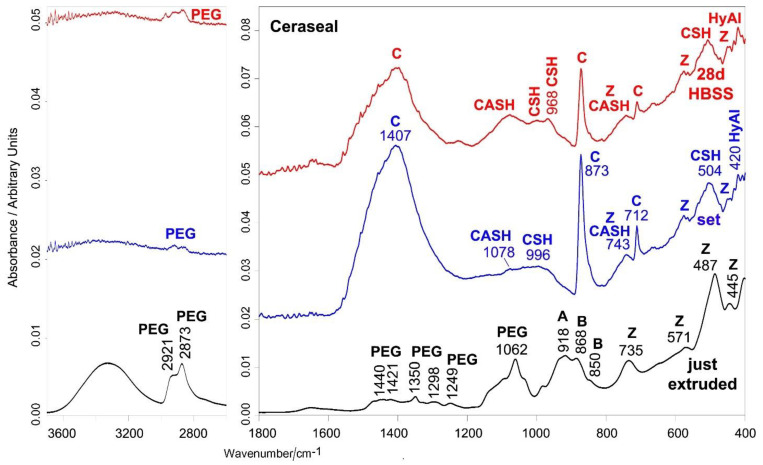
Average IR spectra recorded on just extruded Ceraseal (black) as well as on the surface of set disks before (blue) and after aging in HBSS for 28 days (red). The bands assignable to polyethylene glycol (PEG), alite (tricalcium silicate) (A), belite (dicalcium silicate) (B), monoclinic zirconia (Z), calcite (C), hydrated tricalcium aluminate (HyAl), CSH and CASH phases are indicated.

**Figure 3:**
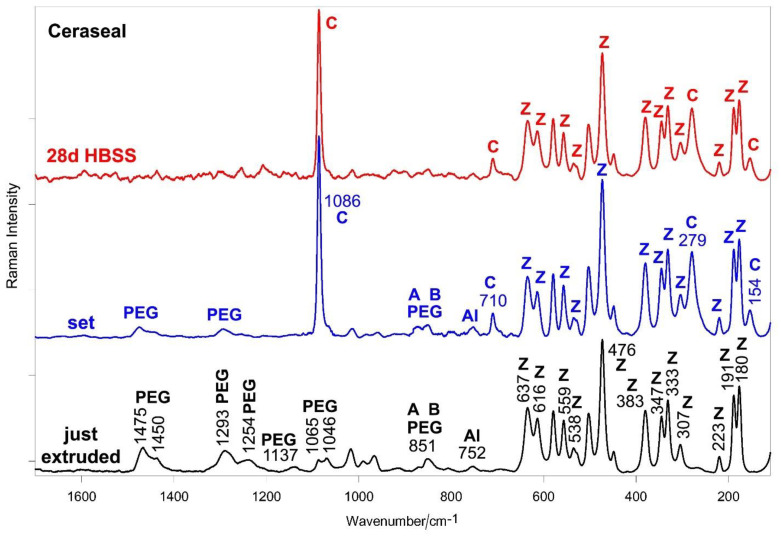
Average micro-Raman spectra recorded on just extruded Ceraseal (black) as well as on the surface of set disks before (blue) and after aging in HBSS for 28 days (red). The bands assignable to polyethylene glycol (PEG), tricalcium silicate (alite) (A), dicalcium silicate (belite) (B), tricalcium aluminate (Al), monoclinic zirconia (Z) and calcite (C) are indicated. PEG component was not indicated in the Material Safety Data Sheet.

Figure 2 and Figure 3 show the average IR and micro-Raman spectra recorded on just extruded Ceraseal as well as on the surface of set disks before and after aging in HBSS for 28 days.

The IR spectrum of just extruded Ceraseal (Figure 2) shows the bands of an organic component only generically declared by the manufacturer as a thickening agent. The IR spectrum would suggest that it could be polyethylene glycol (PEG) of high molecular weight (i.e., higher than 10,000 Da) [24]. The IR bands assignable to alite (tricalcium silicate), belite (dicalcium silicate) [25,26] and monoclinic zirconia [27] were observed as well, in agreement with the composition declared by the manufacturer. Tricalcium aluminate was not detected, since its (weak) marker band at about 750 cm^−1^ [28] is overlapped with the stronger spectral feature of zirconia at 736 cm^−1^. The Raman spectrum of just extruded Ceraseal (Figure 3) confirms the presence of PEG [29], calcium silicates [30,31] and monoclinic zirconia [27,32]; tricalcium aluminate [33,34] was detected as well.

The IR spectrum of the set sample (Figure 2) is dominated by the bands of calcite [35,36,37]. Bands assignable to CaSi particles hydration are detected around 1000 cm^−1^ and at 504 cm^−1^ (calcium silicate hydrate (CSH) gel phase) [38], at 1078 cm^−1^ (calcium aluminosilicate hydrate (CASH) gel phase) [28], 743 cm^−1^ (symmetric stretching vibrations of Si-O-Si (Al) bridges in the Si-O-Si (Al) ring structure of highly polymerized silicates) [39] and 420 cm^−1^ (hydrated tricalcium aluminate) [38].

The micro-Raman spectrum (Figure 3) confirms the presence of calcite [40,41] as the prevailing phase. In both IR and Raman spectra, the PEG and zirconia components were still detected.

After 28 days of aging in HBSS, the main phase remained calcite; CaSi particles hydration proceeded and the IR bands at 1078 and 968 cm^−1^ (Figure 2) became the most prominent spectral features in the 1200–900 cm^−1^ range; the former band suggests the prosecution of CSH chain polymerization and replacement of Al by Si, the latter an increase in the Ca/Si ratio [28]. The bands of the PEG component were detected as weak spectral features (CH stretching at 2921–2873 cm^−1^). The micro-Raman spectrum (Figure 3) is dominated by the bands of calcite and zirconia.

#### 2.5.2. NeoSealer Flo

**Figure 4:**
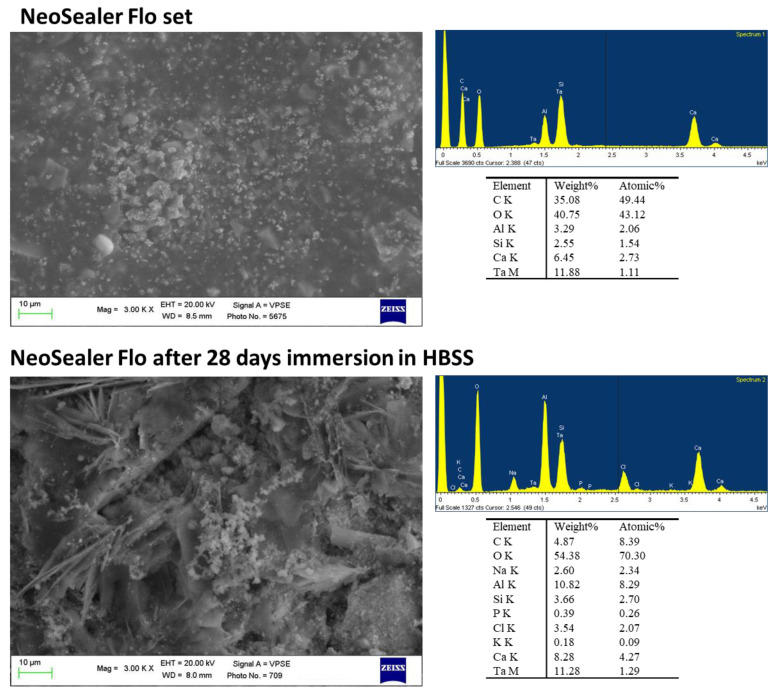
ESEM images at 3000× of NeoSealer Flo before and after immersion in HBSS. The set sample was characterized by a homogeneous surface with granules of different sizes and shapes. EDX analyses revealed the constitutional elements of the sealer, namely Ta, Ca, Si and Al. After 28 days immersion in HBSS, ESEM images revealed a less uniform surface with an irregular layer, characterized by needle-like structures and granules that were agglomerated in larger irregular structures. EDX microanalysis revealed an increase in Ca and Al, stability of Si and Ta and the appearance of P.

ESEM images at 3000× revealed a homogeneous surface with granules of different sizes and shapes (Figure 4). The smaller granules (less than 1 µm) are spread on the whole surface. Larger quadrangular structures (sizes between 2 and 10 µm) were not uniformly distributed. EDX analyses revealed the constitutional elements of the sealer, namely tantalum (Ta) (used as radiopacifier), Ca, Si and Al (from CaSi and calcium aluminates).

After 28 days immersion in HBSS, ESEM images at 3000× revealed a less uniform surface with an irregular layer.

The layer was characterized by long needle-like structures (the length of these needles ranged from 10 to 30 µm) and granules agglomerated in larger irregular structures (ranging from 2 to 5 µm).

EDX microanalysis revealed an increase in Ca and Al, stability of Si and Ta (slight increase) and the appearance of P and Na, K, Cl (from HBSS).

**Figure 5:**
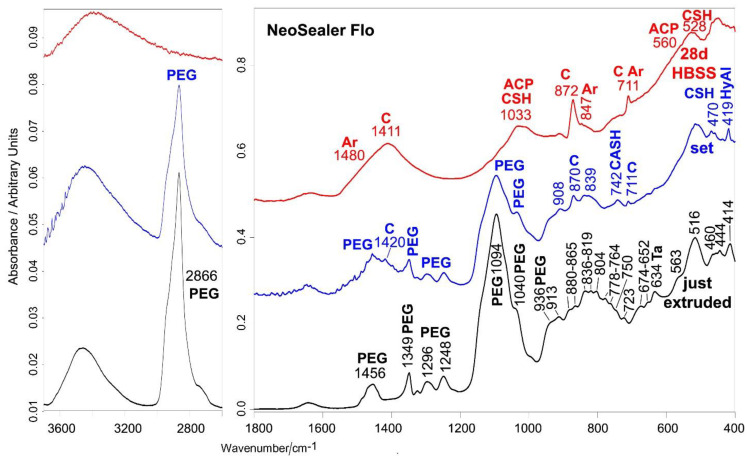
Average IR spectra recorded on just extruded NeoSealer Flo (black) as well as on the surface of set disks before (blue) and after aging in HBSS for 28 days (red). The bands assignable to polyethylene glycol (PEG), tantalite (Ta), calcite (C), aragonite (Ar), hydrated tricalcium aluminate (HyAl), amorphous calcium phosphate (ACP), CSH and CASH phases are indicated. With regard to the 950–400 cm^−1^ range, band assignments to calcium silicates and aluminates are reported in Table S1, Supplementary Material. PEG and grossite were not reported by the manufacturer in the Material Safety Data Sheet.

**Figure 6:**
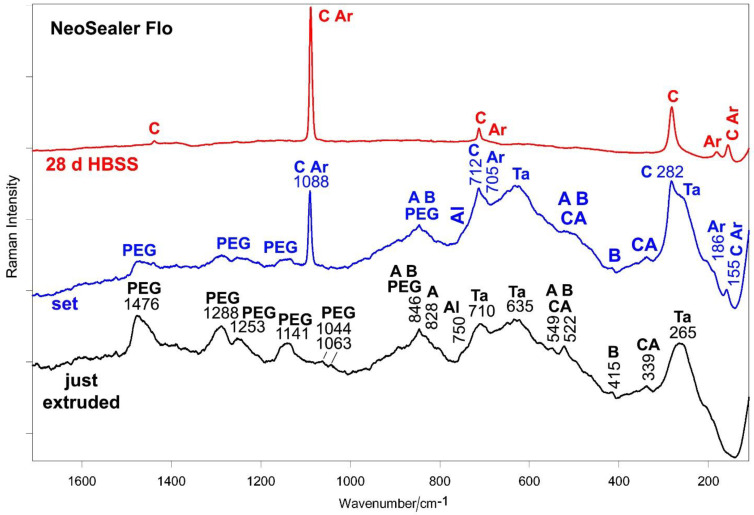
Average micro-Raman spectra recorded on just extruded NeoSealer Flo (black) as well as on the surface of set disks before (blue) and after aging in HBSS for 28 days (red). The bands assignable polyethylene glycol (PEG), tricalcium silicate (alite) (A), dicalcium silicate (belite) (B), tricalcium aluminate (Al), calcium monoaluminate (CA), calcite (C), aragonite (Ar) and tantalite (Ta) are indicated.

Figure 5 and Figure 6 show the average IR and micro-Raman spectra recorded on just extruded NeoSealer Flo as well as on the surface of set disks before and after aging in HBSS for 28 days.

The IR spectrum of the just extruded sealer (Figure 5) showed the presence of an organic component not declared by the manufacturer in the Material Safety Data Sheet; on the basis of the IR spectrum, it is presumably PEG of low molecular weight (i.e., below 1000 Dalton) [24] The IR spectral range below 1000 cm^−1^ is dominated by the bands assignable to silicate and aluminate phases (the latter prevalently as calcium monoaluminate and grossite) [25,26,42]. Grossite was not declared by the manufacturer in the Material Safety Data Sheet. Detailed assignments are given in Table S1, Supplementary Material. Tantalite is revealed by the IR band at 634 cm^−1^ [43], which also has a contribution from AlO_6_ octahedra.

The micro-Raman spectrum of the just extruded sealer (Figure 6) confirmed the nature of the organic component [29], as well as the presence of tantalite [44], calcium silicates and aluminates; among aluminates, calcium monoaluminate and tricalcium aluminate were detected [30,31,33,34,42,45]. For this sealer, setting induced the formation of a calcium carbonate component; the bands of the organic phase weakened in both IR and micro-Raman spectra (Figure 5 and Figure 6). Micro-Raman analysis (Figure 6) showed the presence of both calcite (prevailing phase, i.e., the most stable) and aragonite polymorphic forms [40].

Changes in the relative intensity and shifts of the silicate and aluminate bands in both IR and Raman spectra (Figure 5 and Figure 6) may be ascribed to sealer hydration, according to previous studies [18,38,39]. In particular, the above assigned IR bands at 742 and 419 cm^−1^ (Figure 5) were detected.

Upon aging in HBSS for 28 days, the IR bands of PEG were no longer detected (Figure 5); in particular, its strongest band at 1094 cm^−1^ disappeared, making visible the CSH band [28,39] at 1030 cm^−1^, assignable to the Si-O stretching in aged set Portland cements [36]; the CSH phase was responsible for the increase in intensity of the 450 cm^−1^ band, as previously reported [18,38]. The IR spectrum of the sample immersed in HBSS for 28 days is dominated by the bands of calcite, which appeared even more prominent than in the set sample, suggesting that calcium carbonate deposition proceeded upon aging. The IR spectral feature that reveals signs of calcium phosphate deposition is the broadening around 560 cm^−1^, where the bending mode of amorphous calcium phosphates is reported to fall [46]. This phase could also contribute to the band at 1030 cm^−1^ since in this range PO_4_^3−^ stretching modes are reported to fall. The calcium phosphate deposit is very thin, so that its Raman marker band at 960 cm^−1^ was never detected in the Raman spectra (Figure 6), which showed only the bands of calcium carbonate. This result confirms that the latter is the prevailing phase and suggests that the deposit was thick enough to mask the bands of the material underneath, which were no longer detected. Raman spectroscopy allowed us to clarify that a small amount of aragonite was also present.

#### 2.5.3. AH Plus Bioceramic

**Figure 7:**
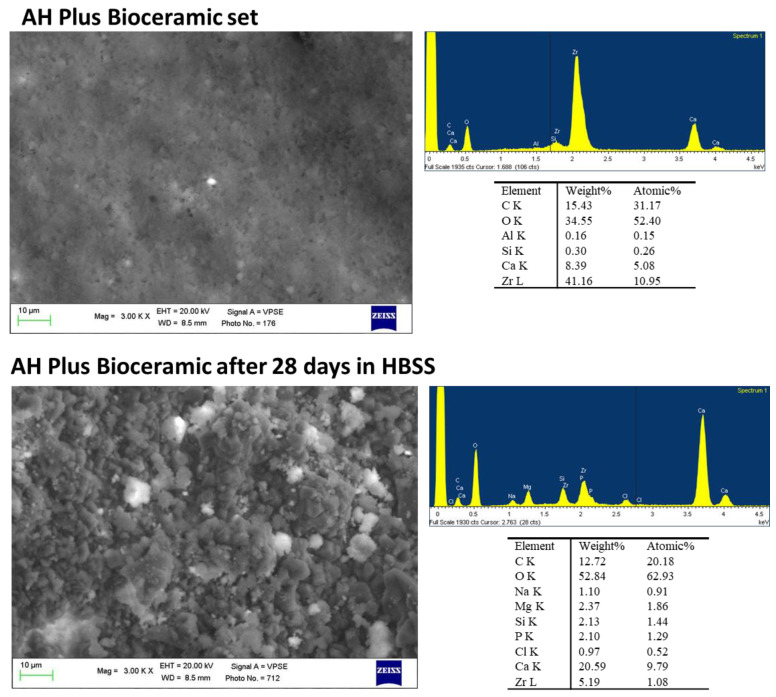
ESEM images at 3000× of AH Plus Bioceramic before and after immersion in HBSS. ESEM on the set sample showed a uniform surface with few irregularities. EDX revealed constitutional elements of the sealer, namely Zr (the radiopacifier) Ca, Si and traces of Al. The surface was covered by a vast layer after 28 days in HBSS. The layer was composed of globular and cubic-shaped structures. EDX analysis revealed a slight increase in Si, a marked increase in Ca, the decrease in Zr, the appearance of P, Na, Cl and Mg.

ESEM images at 3000× of the set sample revealed a uniform surface with few irregularities (Figure 7). EDX analyses revealed the constitutional elements of the sealer, namely Zr (the radiopacifier) Ca, Si and traces of Al. After 28 days in HBSS, the surface was completely covered by a uniform layer, composed of globular structures (range 5–10 µm) and some cubic-shaped structures (range 5–10 µm). EDX analysis revealed a marked increase in Ca, the decrease in Zr and the appearance of P, Na, Cl and Mg (from HBSS).

**Figure 8:**
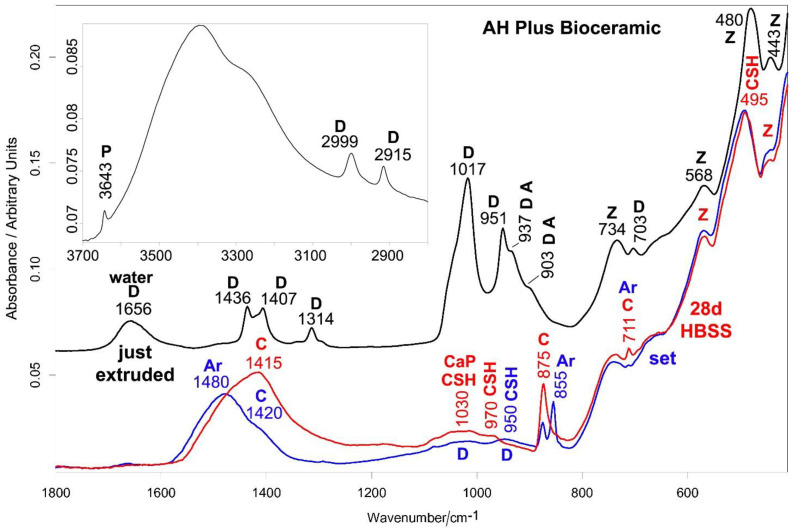
Average IR spectra recorded on just extruded AH Plus Bioceramic (black) as well as on the surface of set disks before (blue) and after aging in HBSS for 28 days (red). The inset shows the 3700–2800 cm^−1^ spectral range of the just extruded sealer. The bands assignable to portlandite (P), dimethyl sulfoxide (D), tricalcium silicate (alite) (A), monoclinic zirconia (Z), CSH phase, calcite (C), aragonite (Ar) and calcium phosphate (CaP) are indicated. Band assignable to water (1656 cm^−1^) was also detected in the just extruded sealer.

**Figure 9:**
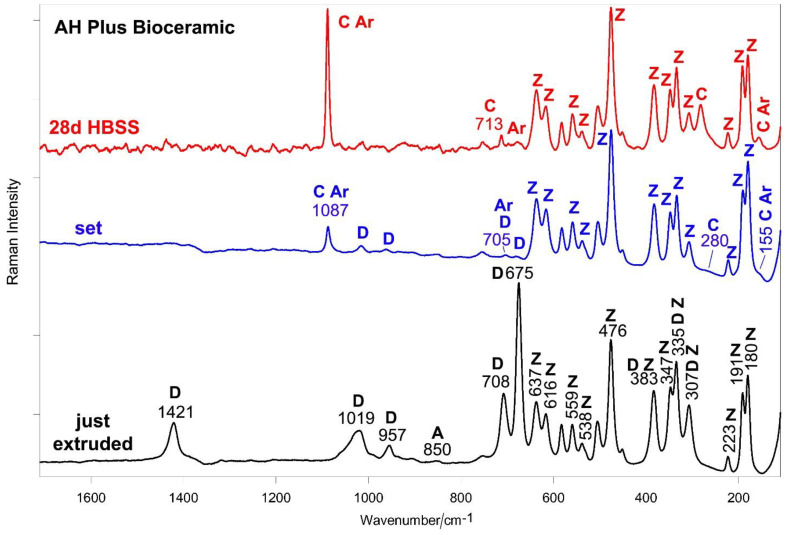
Average micro-Raman spectra recorded on just extruded AH Plus Bioceramic (black) as well as on the surface of set disks before (blue) and after aging in HBSS for 28 days (red). The bands assignable to dimethyl sulfoxide (D), tricalcium silicate (alite) (A), monoclinic zirconia (Z), calcite (C) and aragonite (Ar) are indicated.

Figure 8 and Figure 9 show the average IR and micro-Raman spectra recorded on just extruded AH Plus Bioceramic as well as on the surface of set disks before and after aging in HBSS for 28 days. The IR spectrum of just extruded AH Plus Bioceramic (Figure 8) is dominated by the bands of dimethyl sulfoxide [47]; the bands of monoclinic zirconia dominate the spectral range below 750 cm^−1^ [27]. No bands assignable to lithium carbonate [43] were detected, due to its low content (<0.5% *w*/*w* according to the MSDS). Tricalcium silicate (alite) is revealed by the shoulders at 937 and 903 cm^−1^ [25,28]. The band at 3643 cm^−1^, assignable to Ca(OH)_2_ (portlandite), is due to a partial hydration of the silicate component.

The micro-Raman spectrum of just extruded AH Plus Bioceramic (Figure 9) shows the bands of dimethyl sulfoxide [48,49] and monoclinic zirconia [27,32]. Tricalcium silicate was revealed through the broad band at about 850 cm^−1^ [30,31,45]. No lithium carbonate [43] was detected.

In the Raman spectrum of the set sample (Figure 9), the bands of DMSO decreased in intensity, but did not disappear; the IR spectrum has to be interpreted accordingly (Figure 8). Therefore, the IR bands at about 1030 and 950 cm^−1^ have a contribution from DMSO, although they underwent a significant broadening upon sealer setting. Moreover, they may be assigned to the silicate component. Actually, the band at 950 cm^−1^ may be ascribed to the formation of the CSH phase [36,39] together with the component at about 1030 cm^−1^ as well as the shift and broadening of the band at 480 cm^−1^ due to the contribution of its Si-O-Si bending (reported at about 500 cm^−1^) [39]. Both IR and Raman spectra (Figure 8 and Figure 9) revealed that carbonation of the sealer occurred, with formation of calcite and aragonite polymorphs of calcium carbonate [29,35,36,37,40].

IR spectra (Figure 8) revealed that upon aging in HBSS the prevailing phase is calcite, with low amounts of aragonite; as expected, the most stable polymorph (i.e., calcite) becomes prevalent upon aging for 28 days and evidently aragonite transformed into calcite [40]. By normalizing the spectra to the intensity of the zirconia band at about 735 cm^−1^, only a slight strengthening in the 1030 cm^−1^ spectral profile (where PO_4_^3−^ stretching of calcium phosphates is reported to fall) was observed, whilst no significant increases in the 560–600 cm^−1^ bands (where PO_4_^3−^ bending modes fall) were detected. These trends indicated minor amounts of calcium phosphate phases. The IR band at 950 cm^−1^ shifted to 970 cm^−1^ due to the release of DMSO (whose bands were no longer detected in Raman spectra, Figure 9) and cement maturation [36]. Raman spectra (Figure 9) confirm the presence of calcite and aragonite (the latter in lower amounts) and no detectable amounts of calcium phosphates; actually, the Raman marker band of this phase at about 960 cm^−1^ was not detected.

## 3. Discussion

The study investigated a series of chemical–physical properties of three recent premixed bioceramic sealers. These materials can be used to deal with complex endodontic cases when a traditional sealer might not ensure a stable seal, such as in presence of wide and wet root apices.

Our data showed the setting times of all premixed bioceramic sealers were longer than those reported by the manufacturers, while the setting times of AH Plus were similar to those reported in the literature [50,51].

We found that environmental moisture used to achieve the setting reaction of sealers induced longer setting times than those reported by manufacturers. A similar behavior is reported in a previous paper on IRoot, the first marketed premixed bioceramic sealer, which showed longer sealer setting time with increasing content of humidity [52]. Clinicians should therefore consider waiting longer to ensure the complete material setting before tooth reconstruction. We must underline that it is difficult to clinically assess the suitable humidity of the root canal and no protocols are currently reported to provide a stable and reproducible setting of the sealers.

In order to penetrate into dentinal tubules and to seal complex anatomies, such as isthmuses, secondary canals and apical deltas, the sealer needs to possess high flowability and adequate thickness. In our study, all premixed bioceramic sealers fulfilled the ISO specifications and the results were close to those reported by manufacturer declarations. Ceraseal, in particular, revealed a flowability comparable to AH Plus, which could provide a great advantage in cold obturation techniques, where highly flowable sealers are often recommended. AH Plus flow and film thickness were similar to those previously reported [53].

Concerning our results on radiopacity, all bioceramic sealers revealed lower values when compared to AH Plus. It should be noted that the radiopacity values of the bioceramic-based materials (from 5.5 mm Al of NeoSealer Flo to 8.6 mmAl of AH Plus Bioceramic) were higher than those reported in previous studies where the same procedures were used for bioactive powder-liquid sealers, including Bioroot RCS (5.3 mmAl) [50], Tech Biosealer Endo [3] or paste-to-paste materials, such as MTA Fillapex (4.6 mmAl) [54]. NeoSealer Flo showed significantly lower values, most likely attributable to the presence of tantalite instead of zirconium oxide. No bismuth oxide was included, for the potential tooth discoloration issues and for the toxicity of bismuth when placed close to the periapical tissues [10,15,54]. According to the manufacturer, AH Plus Bioceramic sealer contains approx. 70% zirconium oxide, Ceraseal possesses approx. 50% zirconium oxide, while NeoSealer Flo approx. 40–50% tantalum pentoxide. The significantly higher radiopacity values (11.9 mmAl) of AH Plus may be related to the marked different composition of this sealer, showing higher percentages of calcium tungstate and zirconium oxide radiopacifying agents [50].

All the tested bioceramic sealers showed to be biointeractive materials able to leach calcium ions and to alkalize the soaking water. It is known that calcium ions are strong extracellular signals for mineralizing cells, such as osteoblasts [6,55]. This property is important for materials that should seal the periapical space in the presence of bone defects.

Interestingly, despite the low amount of CaSi (5–15%) in its composition, AH Plus Bioceramic showed a high cumulative calcium release.

Ceraseal provided the highest values of calcium release and alkalizing activity when compared to both NeoSealer Flo and AH Plus Bioceramic. These values were significantly lower than those reported for other materials that were tested under similar experimental conditions, such as Totalfill BC Sealer [56], BioRoot RCS [50] and Neo MTA Plus [57]. This behavior may be attributable to the different percentages of calcium silicates and calcium aluminates in the analyzed materials. Ceraseal and NeoSealer Flo included calcium aluminates in addition to tricalcium and dicalcium silicates, while lower percentages of tricalcium silicate are included in AH Plus Bioceramic sealer, as spectroscopically revealed by the lower relative intensity of the IR and Raman bands of the latter phase with respect to zirconia (Figure 2 versus Figure 8 and Figure 3 versus Figure 9).

The percentage of bioactive CaSi particles in their composition influenced the biointeractive properties of the sealers. Indeed, numerous new biomaterials and scaffolds were developed by increasing the CaSi powder content to enhance the biointeractive and biological properties when applied in periapical bone defects [58,59], or to achieve pulpal revascularization procedures [60,61]. Similarly calcium aluminates (introduced in CaSi-based cement for endodontics to reduce the setting time [62]) showed high Ca^2+^, OH^−^ and Al(OH)_4−_ release [62].

Biointeractivity (release of biologically relevant ions) is related to the high open pore volume which forms an internal network of water-filled pores providing a large surface area involved in the leaching process [14,63]. The ion release depends on the nature of the network structure of the sealer responsible for water absorption and solubility as well as the permeability of the material to water diffusion [14,63].

Therefore, sealers with high open pore volume can absorb more water and consequently show higher solubility and potentially higher ion release (compatibly with their content of reactive CaSi particles). For this reason, NeoSealer Flo and AH Plus Bioceramic showed higher solubility, open pore volume and water absorption. However, a higher solubility of the material tested in water in vitro does not directly indicate a detrimental effect in vivo, as nucleation of apatite and carbonate may compensate and reduce the sealer behavior. It has been demonstrated that the solubility of MTA-like materials is lower when immersed in a medium containing serum protein [64].

Our ESEM-EDX analyses evidenced a layer rich in Ca and C with limited P peaks on the material surface after immersion in HBSS. Apatite nucleation ability was low. In some cases, such as in Ceraseal samples, P was not detected by EDX microanalysis. A possible explanation could be attributable to the Zr and P peaks overlapping, which could mask the presence of P on their surface [65]. This overlapping has also been found in AH Plus Bioceramic, where the presence of P could be underestimated due to the presence of Zr in the material composition.

Previous studies have reported that calcium ions supplied by the rapid dissolution of portlandite (Ca(OH)_2_) and by the cement matrix may react with environmental carbonate ions to form a calcium carbonate superficial layer [19,38,66]. 

Apatite-like and calcite crystals were found on set Portland cement exposed to phosphate solution and CO_2_, and apatite-like phase formation on carbonated substrates [18,63,66]. In previous studies, the calcite crystalline form of calcium carbonate showed good biological activity [18,63,67,68]. Soluble Ca(OH)_2_ crystals allow the nucleation of calcium carbonate polymorphs and/or metastable calcium salt crystals that readily transform into the stable calcite phase in water [18,68]. Calcium carbonate may precipitate at the surface and in the sealer paste porosity, forming a protective layer of calcium carbonate [14,69,70], which can be useful in infected periapical areas. The protective calcium carbonate layer constrains or reduces the ionic diffusion from sealer bulk, reducing its degradation/solubility [14,69,70] and improving the endodontic seal.

In our study IR and micro-Raman spectroscopy allowed to detect the formation of calcium carbonate (as calcite and, in some cases, aragonite) on the surface of all set sealers. This phase strongly influenced the behavior of the materials in the aging tests, i.e., the nature of the phase nucleated upon immersion in HBSS.

The formation of a thin layer of a calcium phosphate phase was detected only on AH Plus Bioceramic and NeoSealer Flo. Ceraseal did not show any calcium phosphate deposits despite its highest calcium release among the tested sealers. This result may be explained by considering that upon setting, this sealer formed the highest relative amount of calcite, as suggested by the highest relative intensity of the Raman and IR bands of this phase (Figure 2 versus Figure 5 and Figure 8, and Figure 3 versus Figure 6 and Figure 9), further strengthening the idea that the material behavior in HBSS was strongly affected by the previous setting conditions.

Nevertheless, these data reveal low/no apatite nucleation on the sealers surface. Possible reasons for low apatite nucleation ability may be related to the low amount of CaSi in the composition of the tested sealers (15% in AH plus Bioceramic, 35% in Neosealer Flo, 20–40% in Ceraseal).

We highlight that a low amount of CaSi in the sealer formulation means lower silanol (Si-OH) functional groups, necessary for the apatite nucleation. It has been shown that the deprotonation of Si-OH at alkaline pH results in the formation of SiO-negative groups [63,71,72], being able to induce a heterogeneous nucleation of calcium phosphates/apatite by bonding calcium ions from the silica-rich mineral particles [17,63,71,72].

As reported by Gandolfi et al. [17], the formation of CaP apatitic precursors is linked to both the ability to release mineralizing ions and the presence of functional groups able to bind ions and trigger the nucleation of apatite.

An additional reason for low apatite nucleation detected on the tested sealers can be attributable to the carbonation processes that may occur during the setting of the materials.

Interestingly, other premixed bioceramics such as Totalfill BC Sealer, revealed a markedly higher nucleation activity in a similar experimental set-up [58]. Indeed, Totalfill BC Sealer demonstrated higher calcium release, alkalization activity, in vitro solubility and apparent porosity [55,73]. The reason for such different behavior could be that Totalfill BC Sealer contained monobasic calcium phosphate in its formulation and a higher content of tricalcium and dicalcium silicates (approx. 50% of the formulation). It has been widely demonstrated that the association of calcium phosphate with hydraulic calcium silicates significantly improved apatite nucleation of the materials [13,19,63].

AH Plus demonstrated a thin CaP deposit despite the low/negligible amount of calcium ions released. This result agreed with previous studies [17,50], where sparse calcium phosphate deposits were detected on AH Plus after immersion in HBSS.

A limitation of this study may be the lack of ex vivo experiments regarding the osteoinductive and proangiogenic properties of the studied sealers.

Only a recent in vitro study on AH Plus Bioceramic sealer tested with human periodontal ligament stem cells [74] reported a similar cytocompatibility but lower mineralization potential when compared to another bioceramic premixed sealer (Endosequence BC Sealer) [74].

An animal model could validate the biointeractive properties found for the tested root canal sealers.

## 4. Materials and Methods

The control paste-to-paste sealer was prepared according to the manufacturer indications. The premixed sealers were ready to be used as specified by the manufacturers. The main components of the materials are listed in Table 6.

Ceraseal is a premixed calcium silicate-based bioceramic sealer and includes, as bioactive components, tricalcium silicate (20–30%), dicalcium silicate (1–10%) and tricalcium aluminate (1–10%). The radiopacifier used is zirconium dioxide and constitutes approx. 45–50% of the composition. Some traces of thickening agents are reported by the manufacturer.

NeoSealer Flo is a premixed bioceramic sealer and includes, as bioactive components, tricalcium silicate (<25%), calcium aluminate (<25%), dicalcium silicate (<10%), grossite (<6%) and tricalcium aluminate (<5%). Tantalite is the radiopacifier and constitutes approx. 50% of the formulation. Traces of calcium sulfate are also reported (<1%).

AH Plus Bioceramic is a premixed bioceramic sealer mostly composed of zirconium dioxide as a radiopacifier (50–70%) and tricalcium silicate (10–15%) as a bioactive component. Dimethyl sulfoxide (DMSO) and traces of lithium carbonate and thickening agents are also reported by the manufacturer.

### 4.1. Initial, Final Setting Times and Radiopacity

Samples were compacted into a mold (10 mm diameter, 2 mm thickness; n = 3 per group) and stored at 37 °C and 99% relative humidity. The initial and final setting times were measured by evaluating the absence of indentation caused by Gillmore needles (ASTM C 226-07 Standard Specification for Air-Entraining Additions for Use in the Manufacture of Air Entraining Hydraulic Cement) with the following modifications performed in accordance with a previous investigation [63]. Ten grams of samples were used instead of 650 g and the physiological temperature of 37 °C instead of 25 °C. The initial Gillmore tip (113.4 g weight and 2.12 mm diameter) and the final Gillmore tip (453.6 g weight and 1.06 mm diameter) were used on the sealer paste.

Radiopacity was tested in accordance with ISO 6876/2012. The materials were compacted into molds (10.0 mm diameter, 1.0 mm height; n = 3 per group). The setting reaction was achieved in 99% relative humidity. Completely set samples were demolded and radiographed using a radiographic unit (Myray Cefla, Imola, Italy) with a reference aluminum step wedge (60 mm long, 10 mm wide thickness varying from 2 to 6 mm in 1 mm increments). The target–film distance was approx. 30 cm with the sample at 3 cm from the surface of the radiographic tube, 0.13 s exposure at 70 kV and 8 mA [56]. The film (Kodak dental film, Eastman Kodak Company, Carestream Health Inc., Rochester, New York, NY, USA) was processed (automatic developer, 4 min at 30 °C) and scanned (Epson Perfection V750 PRO, Jakarta, Indonesia). The radiographic density (colour intensity) data were converted (software ImageJ, Wayne Rasband, National Institutes of Health (NIH), Bethesda, MD, USA) into aluminum step wedge equivalent thickness (mm Al). A suitable sealer should possess radiopacity values equal to or higher than 3 mmAl.

### 4.2. Sealer Flow and Film Thickness

Sealers flow was measured according to ISO 6876/12. The material (0.05 mL ± 0.005) was dispensed onto one glass plate. After 3 min (180 s ± 5), a second glass plate was placed centrally on top of the material and an additional mass (100 g ± 2) was centrally located on the plate (total mass was 120 g ± 2). Ten minutes after the mixing procedure, the weight was removed from the upper glass plate, and the major and minor diameters were measured with a calibrated ruler in mm. This test was replicated three times per material and repeated if discrepancies between minor and major diameters were present. As described in ISO 6876/12, the disk diameter should not be less than 1.7 mm for materials used with gutta-percha points.

Film thickness was measured as described in ISO 6876/12. Briefly, the thickness of 2 glass plates (25 mm length, area of approx. 625 ± 50 mm^2^) was measured using a micrometer. Sealers were placed between two glass plates. After 3 min (180 ± 5 s), a load of 150 N was vertically applied, and the sealer completely filled the area between the glass plates. Ten minutes after the sealer placement, the thickness of the two glass plates and the film of the sealer was measured using a micrometer. Sealer thickness was obtained by subtracting the total thickness and the thickness of the 2 plates previously recorded. The test was repeated 3 times for each material. As described in ISO 6876/12, film thickness should not be more than 50 µm for sealers used with other obturation materials.

### 4.3. Solubility, Water Absorption, Apparent Porosity

Materials compacted into molds (1.0 mm diameter, 1.6 mm height; n = 6 samples for each material) were placed at 37 °C and 99% relative humidity for a period of 100% longer than the final setting time [56]. Samples were demolded, weighed to determine the initial mass (I), immediately immersed vertically in 20 mL of distilled water and placed at 37 °C for 24 h [13,14,51,63]. The mass whilst suspended in water (S) was determined. The samples were then removed from water, the excess water from the surface of each sample was removed using a moistened filter paper and the saturated mass (M) was recorded. Finally, the samples were dried at 37 °C until the weight was stable, and the final dry mass (D) was recorded. Open pore volume (VOP = M − D, in cm^3^), impervious portion volume (VIP = D − S, in cm^3^) and apparent porosity (P = [(M − D)/V] × 100, in percentage) were calculated following Archimedes principle. Water absorption (A = [(M − D)/D] × 100) and solubility (S = [(I − D)/D] × 100) were calculated as percentages of the original weight. Each weight measurement was repeated three times using an analytical balance (Bel Engineering series M, Monza, Italy) and determined to the nearest 0.001 g. Mean values of the measures were reported.

### 4.4. Alkalizing Activity and Calcium Release

Samples were compacted into a mold (8 mm diameter, 1.0 mm height; n = 6 per group) and placed at 37 °C and 99% relative humidity for a period of 100% longer than the final setting time [56]. Then, the materials were demolded and immersed in 10 mL of deionized water (pH 6.8) in polypropylene sealed containers and stored at 37 °C. [13,14,50,57]. The soaking water was replaced at each endpoint (3 h and 1, 3, 7, 14, 28 days) and analyzed for pH and calcium content under magnetic stirring at room temperature (24 °C). A potentiometric method using a multiparameter laboratory meter (inoLab 750, WTW, Weilheim, Germany) was used. For pH measurements, a selective electrode (Sen Tix Sur; WTW) was used, while for calcium release, a calcium probe was used (calcium ion electrode, Eutech Instruments Pte Ltd., Singapore). An ionic strength adjuster was added (4 mol/L KCl; WTW) for calcium release measurements.

### 4.5. Surface Characterization and CaP Nucleation—ESEM-EDX, IR, Micro-Raman

Samples were compacted into a mold (1.0 mm diameter, 2.0 mm height) to reach the final setting time at 37 °C and 99% relative humidity. Then, the samples were demolded, immersed upright in 20 mL of Hanks balanced salt solution (HBSS, Lonza, Verviers, Belgium) and stored at 37 °C for 28 days (ISO 23317:2014), the medium was replaced weekly [15,17,56,57]. The surface of set and 28-day aged materials was examined by environmental scanning electron microscopy (ESEM; Zeiss EVO 50, Jena, Germany) with elemental dispersive X-ray microanalysis (EDX; Oxford Instruments, Abingdon, UK), and vibrational IR and micro-Raman spectroscopy.

Operative ESEM-EDX conditions are reported elsewhere [15,17,56,57]; reported images and elemental analyses are representative of each group. IR spectra were recorded in triplicate on a Bruker Alpha Fourier Transform FTIR spectrometer, equipped with a platinum attenuated total reflectance (ATR) single reflection diamond module (penetration depth 2 µm) and a deuterated lanthanum α-alanine-doped triglycine sulfate (DLaTGS) detector; the spectral resolution was 4 cm^−1^.

Micro-Raman spectra were measured on the surface of the fresh samples as well as after aging in HBSS for 28 days. They were obtained using a Jasco NRS-2000C spectrometer with a microscope of 100× magnification. Five spectra at least were recorded on each sample and averaged. All the spectra were recorded in backscattering conditions with 5 cm^−1^ spectral resolution using a 532 nm green diode-pumped solid-state laser (RgBLase LLC, Fremont, CA, USA) with a power of about 5 mW. A 160 K cooled digital charge-coupled device (Spec-10: 100B, Roper Scientific Inc., Sarasota, FL, USA) was used as a detector.

### 4.6. Statistical Analysis

Data were analyzed using Stata 17.1 (StataCorp, College Station, TX, USA). A two-way ANOVA with RM Student–Newman–Keuls post hoc test (*p* < 0.05) was performed for ion release and alkalinizing activity. One-way ANOVA with Student–Newman–Keuls post hoc test (*p* < 0.05) was used for setting times, radiopacity, solubility, water absorption, apparent porosity, flow and film thickness.

## 5. Conclusions

The study results could be summarized as follows:

- The study supports the clinical use of the three bioceramic root canal sealers.
- The premixed bioceramic sealers met the required chemical and physical standards, but open pore volume, water absorption and solubility were higher when compared to conventional epoxy resin-based sealer.
- Clinicians should be aware that a longer setting time may occur with these materials.
- The premixed bioceramic sealers released biologically relevant ions (as Ca^2+^ and OH^−^) which could provide potential benefits when these materials are positioned close to periapical bone defects or extruded over the root apex.
- Apatite nucleation was slight due to carbonation processes that occurred during setting and after aging in HBSS.

## Figures and Tables

**Table 1:** Initial and final setting times (min, mean ± SD; n = 3) and radiopacity (mmAl, mean ± SD; n = 3). Different superscript letters (vertical row) indicate statistically significant differences (*p* < 0.05) among materials.

	Initial Setting Time	Final Setting Time	Radiopacity
Ceraseal	60 ± 5 ^a^	660 ± 60 ^a^	8.0 ± 0.5 ^a^
NeoSealer Flo	480 ± 125 ^b^	1344 ± 60 ^b^	5.5 ± 0.5 ^b^
AH Plus Bioceramic	360 ± 60 ^c^	720 ± 60 ^c^	8.6 ± 0.5 ^b^
AH Plus	470 ± 5 ^b^	1300 ± 60 ^b^	11.5 ± 0.5 ^c^

**Table 2:** Flowability (min, mean ± SD; n = 3) and film thickness (µm, mean ± SD; n = 3) of just extruded sealers. Different superscript letters (vertical row) indicate statistically significant differences (*p* < 0.05) among materials.

	Flowability	Film Thickness
Ceraseal	2.94 ± 0.09 ^a^	70.7 ± 4.0 ^a^
NeoSealer Flo	1.88 ± 0.06 ^b^	128.7 ± 8.1 ^b^
AH Plus Bioceramic	2.38 ± 0.13 ^c^	174.0 ± 5.3 ^c^
AH Plus	2.96 ± 0.10 ^a^	68.3 ± 2.9 ^a^

**Table 3:** Open pore volume (cm^3^, mean ± SD; n = 6), water absorption and solubility (%, mean ± SD; n = 6) of tested sealers. Analyses were performed on set materials (+100% of final setting time). Different superscript letters (vertical row) indicate statistically significant differences (*p* < 0.05) among materials.

	Open Pore Volume	Water Absorption	Solubility
Ceraseal	0.024 ± 0.004 ^a^	11.2 ± 3.6 ^a^	1.02 ± 0.43 ^a^
NeoSealer Flo	0.048 ± 0.003 ^b^	33.0 ± 11.0 ^b^	7.10 ± 3.5 ^b^
AH Plus Bioceramic	0.042 ± 0.011 ^b^	24.8 ± 8.5 ^b^	5.80 ± 1.5 ^b^
AH Plus	0.0030 ± 0.0002 ^c^	1.40 ± 0.20 ^c^	0.80 ± 0.12 ^a^

**Table 4:** Alkalizing activity (mean ± SD; n = 8) of tested materials. The pH of soaking water was measured after immersion of set sealers (+100% of final setting time). Different superscript letters (vertical row) indicate statistically significant differences (*p* < 0.05) among sealers.

	3 h	1 Day	3 Days	7 Days	14 Days	28 Days
Ceraseal	9.51 ± 0.10 ^a^	10.01 ± 0.28 ^a^	9.64 ± 0.34 ^a^	9.01 ± 0.13 ^a^	8.75 ± 0.15 ^a^	8.13 ± 0.16 ^a^
NeoSealer Flo	8.73 ± 0.10 ^b^	8.40 ± 0.04 ^b^	8.23 ± 0.12 ^b^	8.43 ± 0.03 ^b^	8.38 ± 0.06 ^b^	8.17 ± 0.11 ^a^
AH Plus Bioceramic	8.6 ± 0.6 ^b^	9.5 ± 0.5 ^ab^	9.0 ± 0.6 ^a^	8.4 ± 0.5 ^b^	8.4 ± 0.5 ^b^	8.3 ± 0.6 ^a^
AH Plus	7.7 ± 0.2 ^c^	7.4 ± 0.1 ^c^	7.3 ± 0.2 ^c^	7.3 ± 0.2 ^c^	7.2 ± 0.1 ^c^	7.1 ± 0.3 ^c^
Deionized water	7.02 ± 0.17 ^d^	7.28 ± 0.32 ^c^	7.12 ± 0.32 ^c^	7.05 ± 0.35 ^c^	7.12 ± 0.32 ^c^	6.98 ± 0.25 ^c^

**Table 5:** Calcium release (ppm, mean ± SD; n = 8) of tested sealers. Calcium release in soaking water was measured after immersion of set materials (+100% of final setting time). Different letters indicate statistically significant differences (*p* < 0.05) among materials.

	3 h	1 Day	3 Days	7 Days	14 Days	28 Days	Cumulative
Ceraseal	43.73 ± 5.7 ^a^	80.85 ± 10.89 ^a^	89.52 ± 5.68 ^a^	57.53 ± 10.31 ^a^	41.95 ± 4.65 ^a^	38.30 ± 13.18 ^a^	347.45 ± 16.25 ^a^
NeoSealer Flo	40.56 ± 14.4 ^a^	41.37 ± 8.6 ^b^	47.95 ± 19.9 ^b^	36.77 ± 7.2 ^b^	27.25 ± 2.4 ^b^	21.77 ± 2.4 ^b^	205.25 ± 35.4 ^b^
AH Plus Bioceramic	20.5 ± 10.6 ^b^	30.6 ± 10.5 ^b^	67.5 ± 20.5 ^c^	40.5 ± 7.5 ^b^	35.5 ± 10.5 ^ac^	20.58 ± 8.6 ^b^	200.5 ± 80.5 ^b^
AH Plus	1.8 ± 0.8 ^c^	1.9 ± 0.5 ^c^	1.3 ± 0.4 ^d^	2.1 ± 0.2 ^c^	2.5 ± 1.2 ^d^	0.5 ± 0.2 ^c^	1.4 ± 0.5 ^c^
Deionized water	1.6 ± 0.5 ^c^	1.5 ± 0.3 ^c^	1.2 ± 0.1 ^d^	1.1 ± 0.1 ^c^	1.5 ± 0.6 ^d^	0.5 ± 0.2 ^c^	0.7 ± 0.5 ^c^

**Table 6:** Sealer formulation, lot and composition in accordance with manufacturer Safety Data Sheet. Asterisk (*) indicates the components detected by our investigation.

Sealer and Manufacturer	Formulation	Lot	Composition
Ceraseal (MetaBiomed, South Korea)	Premixed	CSL2108201	Zirconium dioxide (45–50%), tricalcium silicate (20–30%), dicalcium silicate (1–10%), tricalcium aluminate (1–10%), thickening agents, polyethylene glycol (PEG) *
NeoSealer Flo (Avalon Nusmile, USA)	Premixed	2020110502	Tantalite (50%), tricalcium silicate (25%), calcium aluminate (25%), dicalcium silicate (10%), tricalcium aluminate (5%), calcium sulfate (1%), PEG *, grossite *
AH Plus Bioceramic (Maruchi, South Korea)	Premixed	KI211103	Zirconium dioxide (50–70%), tricalcium silicate (5–15%), dimethyl sulfoxide (10–30%), lithium carbonate (0.5%), thickening agents (<6%)
AH Plus (Dentsply, Germany)	Paste–paste	2109000972	Paste A: diepoxide, calcium tungstate, zirconium oxide, aerosil, pigment (iron oxide) Paste B: 1-adamantane amine, N,N′-dibenzyl-5-oxa-nonandiamine-1,9, TCD-diamine, calcium tungstate, zirconium oxide, aerosil, silicone oil

## Data Availability

Not applicable.

## Supplementary Materials

The following supporting information can be downloaded at: https://www.mdpi.com/article/10.3390/ijms232213914/s1.
