# Supplementary material for: Chemical-Physical Properties and Bioactivity of New Premixed Calcium Silicate-Bioceramic Root Canal Sealers
Source: Int J Mol Sci. 2022 Nov 11;23(22):13914. doi: 10.3390/ijms232213914 (PMC9692705; doi:10.3390/ijms232213914)
Supplement: Supplementary file 1 [file ijms-23-13914-s001.zip › ijms-2014317-supplementary.pdf]

Supplementary Material

Chemical–Physical Properties and Bioactivity of New Premixed Calcium Silicate–Bioceramic Root Canal Sealers

Fausto Zamparini [1,2], Carlo Prati [1], Paola Taddei [3], Andrea Spinelli [1], Michele Di Foggia [3] and Maria Giovanna Gandolfi [2,*]

[1] Endodontic Clinical Section, School of Dentistry, Department of Biomedical and Neuromotor Sciences, University of Bologna, 40125 Bologna, Italy
[2] Laboratory of Green Biomaterials and Oral Pathology, School of Dentistry, Department of Biomedical and Neuromotor Sciences, University of Bologna, 40125 Bologna, Italy
[3] Biochemistry Unit, Department of Biomedical and Neuromotor Sciences, University of Bologna, 40126 Bologna, Italy
* Correspondence: mgiovanna.gandolfi@unibo.it

Table S1. 950-400 cm^{-1} IR bands wavenumbers and assignments [Hughes…] for just extruded NeoSealer Flo.

Just extruded NeoSealer Flo Wavenumber / cm^{-1}	Assignments
936 sh	PEG, alite, grossite
913	Alite, calcium monoaluminate
880 sh	PEG, belite
865	Calcium monoaluminate, grossite, tricalcium aluminate
836	Calcium monoaluminate, belite
819	Calcium monoaluminate, grossite, tricalcium aluminate
804	Calcium monoaluminate
778	Calcium monoaluminate, tricalcium aluminate
764	Tricalcium aluminate
750 sh	Grossite
723	Calcium aluminate
674	AlO_6 octahedra
652	Calcium aluminate
634	Tantalite, AlO_6 octahedra
563 sh	AlO_6 octahedra
516	Fewly polymerized CxS silicate tetrahedra and AlO_6 octahedra
460	AlO_6 octahedra
444	Fewly polymerized CxS silicate tetrahedra
414	AlO_6 octahedra

sh = shoulder
